# Nosological Strictures

**Published:** 1818-09

**Authors:** T. Parkinson


*For the London Medical and Physical Journal.*

### *Nosological Strictures;* by T. Parkinson, M.D.

"The word FEVER having been suffered to remain a denominating term for disease, has been the silent cause of more misery and mortality among mankind than the sword or the plague."

I AVER that there is not, nor possibly can be, Fever without local inflammation as its sole cause. The identical affection is *inflammation* of some distinguishable GENUS of Solids, of which there are but seventeen in the whole animal system; and these are all comprehended under two distinct ORDERS, viz.

1. PLATYMORPHĪA; πλατυς μορφοω; all the GENERA of Animal Solids excepting Adipose Substance and Glands.

2. SPHARIMORPHĪA; Σφαιρα μορφοω; Adipose Substance and Glands only.

Any one GENUS of Solid in the first ORDER being inflamed to a given degree and extent, the general System will suffer, and its form of suffering will be what has been called TYPHOID FEVER, assuming an intermittent, or a remittent, or a progressive *Type*, according to the degree of *intensity* of local inflammation; and, on the subduction of such local inflammation, the Fever will cease. But, if the Type be progressive, and the disease proceeds to the death of the patient, the local inflammation will be increasing in intensity, and the constitutional suffering will be commensurate, until the local inflammation ends in Gangrene, or the Vital Powers are exhausted by mortal excess of exercise.

This is the true picture of what I have denominated in my Synopsis Zoonosologiæ, HYPERBIŌSIS DIFFUSA, as a CLASSICAL appellation; but, as CLETIC, I have chosen ENYPHANTITIS PLATYNŌDES, meaning Inflammation of Simple Membrane, as Dura-mater, Pia-mater, Pleura, Peritonæum, Fascia, &c.; and, as PHANERIC, I have chosen PARENTHĪTIS, meaning Morbid Excess of Function in an interposing Membrane.

By adding the Specific adjective, denoting Situation, the character of the disease will be shown; and, by adjoining the Typical adjective, denoting the Intensity, a Nosological SYNTAX will be formed, representative of the Character and Violence of the Disease.

EXAMPLES.

PARENTHĪTIS encephalica regressiva,
———————— intermittens,
———————— remittens,
———————— progressiva,
———— thoracica,
———— enteronica,
———— exoterica, with their Types.

Either of the GENERA of Solids in the second ORDER being inflamed to a given degree and extent, the general System will suffer, and its form of suffering will be what has been called *Synocha Fever*, assuming a Regressive or a Progressive Type; and, on the subduction of the local inflammation, the Fever will cease. But, if the Type be progressive, and it proceeds to the death of the patient, the local inflammation will be increasing in intensity, and the constitutional suffering will be commensurate, until the local affection ends in Abscess, or the vital powers are exhausted by mortal excess of exercise.

This is a true picture of what I have denominated, CLASSICALLY, HYPERBIOSIS SPHÆRĪCA.

CLETIC.—HELASMĪTIS SIPHOGYRŌDES. Inflammation of an Arterious Gland.
ENYPHANTĪTIS STEATŌDES. Inflammation of Adipose Substance.
ENYPHANTĪTIS SIPHOGYRŌDES. Inflammation of a Venous Gland—the Liver.

PHANERIC.—ECCRISĪTIS HELASMĪCA. Excess of Function of an Arterious Gland.
MORPHOSĪTIS. Excess of Function of Adipose Substance.
ECCRISITIS ENYPHANTĪCA. Excess of Function of a Venous Gland.

By adjoining the Specific and the Typical adjectives, the character and violence of the disease will be represented.

EXAMPLES.

ECCRISITIS encephalica regressiva. Inflammation of a Gland within the Head, with slight Intensity; therefore, about to retire.

ECCRISITIS encephalica progressiva. Inflammation of a Gland within the Head, with great Intensity; therefore, proceeding to death, and so on through all the Species.

MORPHOSITIS exoterica regressiva. Inflammation of Adipose Substance externally, with slight intensity, and so on.

*Leicester; July* 14, 1818.

